# The Perception of Nursing Professionals Working in a Central Sterile Supplies Department regarding Health Conditions, Workload, Ergonomic Risks, and Functional Readaptation

**DOI:** 10.1155/2022/1023728

**Published:** 2022-04-13

**Authors:** Rosemere Saldanha Xavier, Patrícia dos Santos Vigário, Alvaro Camilo Dias Faria, Patricia Maria Dusek, Agnaldo José Lopes

**Affiliations:** ^1^Local Development Post-Graduation Program, Augusto Motta University Center (UNISUAM), Rio de Janeiro, Brazil; ^2^Rehabilitation Sciences Post-Graduation Program, Augusto Motta University Center (UNISUAM), Rio de Janeiro, Brazil; ^3^Unesulbahia-Integrated Faculties, Salvador, Bahia, Brazil; ^4^Medical Sciences Post-Graduation Program, School of Medical Sciences, State University of Rio de Janeiro (UERJ), Rio de Janeiro, Brazil

## Abstract

**Background:**

The central sterile supply department (CSSD) is wrongly seen as a place in the hospital environment that does not require skills and physical effort, being commonly a hospital sector for the relocation of functionally-readapted professionals. However, CSSD is a work environment that demands professional experience and presents itself as a sector that does not have a healthy work environment. This study aims to evaluate the frequency of comorbidities and functionally-readapted people among nursing professionals allocated to a CSSD and, also, to seek the perception of these professionals about the ergonomic risks and the degree of difficulty to perform activities within a CSSD.

**Methods:**

This is a cross-sectional study that analyzed the opinions of nursing professionals who work in the CSSD of public hospitals in Rio de Janeiro, Brazil. Nurses, nursing technicians and nursing assistants aged ≥18 years were included.

**Results:**

Seventy-two nursing professionals were consecutively evaluated. It was observed that 43 of them (59.7%) had never worked in a CSSD. The most prevalent comorbidity in the present study was chronic rhinosinusitis, observed in more than half of the sample, although it is interesting to note the high frequency of participants with work-related musculoskeletal disorders (WMSD) and repetitive strain injuries (RSI). There is a relationship between previous work in a CSSD and the ability to identify surgical tweezers by visual recognition (*p*=0.031). There is a relationship between the time the participant had previously worked in the hospital and the skill regarding the information contained in the conference folders for preparing the tray surgical procedures (*τb* = −0.34, *p*=0.001).

**Conclusion:**

Almost a third of nursing professionals working in a CSSD are rehabilitated, with a high prevalence of WMSD and RSI. The commitment of managers to an internal health policy aimed at workers is necessary for health promotion.

## 1. Introduction

The central sterile supply department (CSSD) is a hospital sector dedicated to the reception, cleaning/disinfection, preparation, sterilization, storage, and distribution of materials for the entire hospital unit [1]. However, the CSSD is a stigmatized area within the hospital. Initially, the activities of preparation and sterilization of hospital instruments were the tasks of professionals who worked in the operating room, and there was no autonomy or even recognition as a unit [2]. Although the CSSD has full responsibility for the processing of hospital supplies, its recognition as an essential area for the provision of patient care is still small [3, 4].

Historically and nowadays, CSSD is classified as a sector of indirect activities for patient care and considered as a secondary sector [5, 6]. It is a technical support unit responsible for supplying properly processed medical and hospital instruments, with the aim of offering adequate conditions for direct care and assistance to patients. The CSSD work process has its own characteristics and peculiarities to be aimed at nursing professionals [7]. After the material is processed, it is intended to meet the procedures performed in outpatient and inpatient units, especially surgical procedures. Despite the invisibility and little appreciation of the activities carried out in the CSSD to superimpose the activities of other sectors of the hospital, nursing professionals are aware of the importance of their work in patient safety. Considering the benefits of offering patients in the hospital environment without risk to their health regarding processed material and free from microorganisms, CSSD is important for patient safety in surgical procedures. Therefore, all actions involved in the CSSD work process are essential requirements that are enabled in the proper processing of instruments and that contribute to the safety of surgery [8].

Improving the quality and safety of the CSSD work process encompasses the entire processing of surgical instruments, the management of surgical trays, and efforts to ensure that the information is efficient. All these steps are intrinsically linked to the success of the surgery [9]. Avoiding CSSD failures is working for patient safety and, thus, the sector must have a qualified nursing team and employees aware of the importance of the steps in the cleaning processes, preparation of surgical trays, sterilization, and storage [10]. The direct supervision of the nursing professional must occur at all stages of the process, using validated protocols for this [7].

The CSSD is considered a place that does not require skills and physical effort, being commonly a hospital sector for the relocation of functionally-readapted professionals. However, this invisibility is characterized by the lack of knowledge of other health professionals and the activities performed, generating dissatisfaction among professionals working in the CSSD [11]. Functional readaptation is directly linked to the relocation of health professionals to other activities or sectors that do not harm their current health condition. However, CSSD is a work environment that demands professional experience and presents itself as a sector that does not have a healthy work environment due to exposure to chemical agents and contaminants; this contributes to health problems for professionals, whether physical or psychological [12].

Health conditions, workload, physical effort, functional readaptation, and degree of difficulty in performing manual tasks (including identification of surgical tweezers) among nursing professionals working in a CSSD are still poorly known [5, 6, 9, 12]. Thus, the present study sought to evaluate the frequency of comorbidities and functionally-readapted people among nursing professionals allocated to a CSSD and, also, to seek the perception of these professionals about the ergonomic risks and the degree of difficulty to perform activities within a CSSD.

## 2. Materials and Methods

### 2.1. Study Design and Participants

Between June and July 2021, a cross-sectional study was carried out analyzing the opinions of nursing professionals working in the CSSD of public hospitals in Rio de Janeiro, Brazil. Nurses, nursing technicians, and nursing assistants, of both sexes, >18 years old and with the following employment relationships were included: statutory, workers governed by the Consolidation of Labor Laws, and service providers working in the CSSD (both on the day and on the night shift).

The project was approved by the Research Ethics Committee of the Augusto Motta University Center under the number CAAE-45992721.9.0000.5235, and all subjects signed the consent form. The protocol followed the recommendations for research in human beings as per the Declaration of Helsinki.

### 2.2. Assessment Instrument

A questionnaire containing sociodemographic data, health conditions, length of professional experience, and opinions about the skills of nursing professionals (including the preparation of surgical trays) was applied. To this end, we resorted to the construction of questions using the Likert scale, which is a psychometric response scale used particularly in the area of psychology, health, and education, to which the participant responds through a criterion that can be objective or subjective [13, 14]. Usually what one wants to measure is the level of agreement or non-agreement to the statement, usually using five levels of response [14].

### 2.3. Data Analysis

Descriptive analysis was presented in the form of tables and the observed data were expressed by frequency and percentage. The inferential analysis consisted of the following methods: the comparison of professional ability (5 classes) between professionals with and without previous work in the CSSD was assessed using Fisher's exact test; and the association between professional ability and length of time working at the hospital (3 classes) and performance upon reaching the CSSD (5 classes) was analyzed using Kendall's tau-b (*τ*b) correlation coefficient. The criterion for determining the significance adopted was the level of 5%. Statistical analysis was performed using SAS 6.11 software (SAS Institute, Inc., Cary, NC, USA).

## 3. Results

Seventy-two nursing professionals were consecutively evaluated as follows: 13 nurses; 56 nursing technicians; and 3 nursing assistants. Regarding age, the age group between 51 and 60 years predominated (34.7% of the sample). Among the participants, 54 (75%) were female and 35 (48.6%) declared themselves married. In this sample, 42 participants (58.3%) had secondary education and most had a statutory relationship with hospital institutions. The general characteristics of the participants are shown in Table 1.

**Figure 1:**
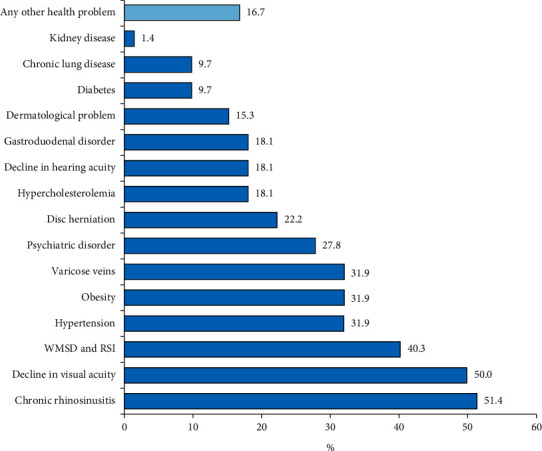
Decreasing distribution of comorbidities in the evaluated sample. WMSD: work-related musculoskeletal disorders. RSI: repetitive strain injuries.

As for the length of service provided to the institutions, it is observed that almost half of the professionals evaluated (47.2%) had between 11 and 20 years of work in the hospital. According to the professional profile of the interviewees, it was observed that 43 of them (59.7%) had never worked in a CSSD. Regarding the relocation of nursing professionals in the CSSD, 22 (30.6%) responded that they came to the sector due to functional readaptation. Data regarding the professional profile of the participants are shown in Table 2. The most prevalent comorbidity in the present study was chronic rhinosinusitis, observed in more than half of the sample, although it is interesting to note the high frequency of participants with work-related musculoskeletal disorders (WMSD) and repetitive strain injuries (RSI), such as shown in Figure 1.

Regarding the structure of the workplace, 43 (59.7%) participants strongly disagreed that the CSSD is a place where light activities that require little physical effort are performed. They also disagreed [*n* = 30 (41.7%)] that the CSSD provides an adequate framework for promoting professional health. Regarding the tasks performed in the CSSD, 40 (55.6%) agreed that they can be considered harmful to the health of the functionally-readapted professional. Regarding skills, 23 (31.9%) participants responded that it was likely to prepare a surgical tray without the use of conference folders. Fifty-four (75%) of the respondents fully agreed that the correct preparation of surgical trays contributes to patient safety during surgery. Thirty-six (50%) participants agreed that CSSD presents a high risk of contamination compared to other hospital sectors. Thirty-four (47.2%) participants responded that it is likely that the use of a sustainable product for the identification of surgical clamps improves their identification. The distribution of participants' opinions regarding health risk, professional skills, and work environment in the CSSD is shown in Table 3.

Additionally, we evaluate some associations.  Table 4 shows a significant association between prior work in CSSD and the ability to identify surgical clamps by visual recognition using Fisher's exact test (*p*=0.031). Observing the distribution of responses, the group of participants with previous work in a CSSD showed a greater propensity to classify “very easy” in the task of identifying tweezers by visual recognition (17.2%) than the group without previous work in a CSSD (0%).  Table 5 shows a significant association between the time the participant had previously worked in the hospital and the ability to use the information contained in the conference folders to prepare the surgical trays using Kendall's *τb* (*τb* = −0.34, *p*=0.001).

## 4. Discussion

The main findings of the present study were that almost 80% of the nursing professionals working in a CSSD have up to 20 years of service in the hospital and almost 60% of them had never previously worked in this hospital sector. Importantly, almost a third of nursing professionals working in a CSSD came to the sector because of functional readaptation. There was a high prevalence of people with WMSD/RSI. Most of these professionals believe that the CSSD is a hospital place where activities that demand a lot of physical effort are performed, which are harmful to the health of the functionally-readapted professional. There was a relationship between previous work in a CSSD and the ability to identify surgical tweezers by visual recognition, and between the time the participant had previously worked in the hospital and the skill regarding the information contained in the conference folders for preparing the tray for surgical procedures.

Among the sociodemographic characteristics analyzed in this study, there was a clear predominance of female nursing professionals. Historically, the art of caring is linked to the figure of the woman, which is reflected in the increase in the workforce in health units, not only in Brazil but also in other countries [12, 15]. By observing the profile of nursing professionals, it was possible to identify a clear predominance of nursing technicians, which suggests that these people have other professions that do not require university education. This highlights the need to encourage the construction of professional growth or even encourage professional growth [16]. As for professional experience, it was observed that almost 60% of the participants had never worked in a CSSD, which characterizes an incorrect conception that the CSSD is a sector for workers who do not have professional experience. According to Bugs et al., the CSSD is a unit that preferably ensures quality care—whether in the inpatient or surgical unit—with hospital instruments being essential elements in this process [17].

Given the invisibility of the CSSD, which unfortunately many managers still believe is a place exclusively for functionally-readapted professionals, almost a third of our sample responded that they came to work in this sector for functional readaptation. In this context, it should be noted that this scenario is changing. It can be said that the movement of professionals to the CSSD and the growing technological advancement of instruments and products used in the sector—which requires professionals with technical knowledge and vast experience—are factors that could have positive repercussions in this scenario. Even though it is classified as indirect care, with routine and repetitive activities, the importance of the work of CSSD professionals is reflected in the delivery of a contamination-free product for hospital use [18]. A recent study showed that CSSD can successfully form closed-loop management of sterilization effectiveness, improve standardized management of sterile article storage in clinical departments, ensure sterile article safety, and reduce the cost of consumption caused due to loss of sterile packaging [19].

The intense workload and frequent exposure to ergonomic risks are the predominant factors for the emergence or worsening of comorbidities, especially WMSDs and RSIs [20]. In the present study, these comorbidities had a prevalence rate of 40%. Nursing professionals are highly exposed to intense and strenuous work, being part of the group of workers who are at risk for occupational diseases and other health problems such as psychiatric disorders [21, 22]. One aspect that needs to be emphasized is that the intense workload and occupational diseases are conditions that contribute to the illness of nursing professionals worldwide. In line with the studies by Costa et al. [11] and Morais et al. [23] and, almost 60% of respondents in our study strongly disagreed that the CSSD is a sector where light activities that require little physical effort are performed. It is noteworthy that more than 40% of our sample disagreed that the CSSD offers an adequate structure to promote the health of professionals due to occupational and ergonomic risks, as they are exposed to chemical and physical agents despite the routine use of personal protective equipment.

Regarding the tasks performed in the CSSD, more than half of the participants agreed that they can be considered harmful to the health of the functionally-readapted professional. This is interesting because, in general, these professionals are of older age, with their retirement approaching, and present health conditions that prevent them from fully exercising their activities. This is reinforced by the fact that the work assigned to nursing professionals requires physical effort, repetitive tasks, and uncomfortable positions [12]. It is noticeable that the role of health professionals in the CSSD goes beyond routine and repetitive work, requiring specific training for qualification in the development of specific skills to work in this sector [11]. This finding is in agreement with the study by Santos et al., which points out that the elimination of unnecessary or redundant instruments in surgical trays can save time, require less operational effort, and bring lower costs to the CSSD, without compromising the surgical procedure and the health of patients [24]. Therefore, simplifying surgical instruments can also significantly reduce sterilization time, decrease ergonomic risks, and lower instrument acquisition costs.

The correct preparation of surgical trays can directly impact patient safety during surgery. In our study, 75% of the participants strongly agreed with this question, which demonstrates the perception of these health professionals regarding the importance of their work. Additionally, we observed a positive association between previous work in a CSSD and nursing professionals' self-declaration about the ability to identify surgical clamps by visual recognition. The World Health Organization cites some worrying situations in patient safety within the hospital environment, including hospital infections and other surgical complications [25]. The CSSD aims to offer patients a microorganism-free material, whose process is carried out in several steps. This is corroborated by the study by Sartelli et al., which shows the need to focus the efforts of all health professionals on the prevention and control of infections [26]. It is worth emphasizing the inverse association between the time the participant previously worked at the hospital and the skill they attribute to the information contained in the conference folders for the preparation of surgical trays. A possible explanation for this paradox is that people with less time working in the hospital are younger and more adapted to new technologies, which allows for greater ease in preparing surgical trays.

Finally, it is important to highlight the importance of strengthening actions aimed at preserving the environment, as the CSSD is a hospital sector that needs to implement these actions. Interestingly, more than half of the participants (52.8%) agreed with these actions, while 47.2% responded that the use of a sustainable product for the identification of surgical clamps is likely. The use of sustainable technological products can be a tool for improving the work process and quality of care. It should also be noted that, with the necessary knowledge and the use of technologies with well-planned actions, it is possible to prevent harm to patients, improving the quality of care provided in health care. However, the need to adhere to these improvements is a key factor for greater patient care [27, 28].

The strength of this study is that it is the first study that addresses in detail the opinions of nursing professionals who work in a CSSD, encompassing professionals from different hospitals and with varied experiences (including the handling of surgical trays). However, some limitations must be pointed out. First, the number of participants was relatively small, although we included professionals from both the day and night shifts and from different age groups. Second, our measurement was based on the application of a questionnaire and, therefore, the use of objective measures is important to confirm our results. Third, we only evaluated the opinion of nursing professionals working in public institutions. Despite these limitations, our results can serve as a starting point for future research with greater numbers of nursing professionals working in CSSD using objective measures to assess safety and the work environment. We also encourage interventional studies aimed at promoting health and facilitating the tasks of these professionals, such as the preparation of surgical trays.

## 5. Conclusions

Our study shows that almost a third of the nursing professionals working in a CSSD are functionally-readapted people, with a high prevalence of WMSD/RSI. Most of them believe that the CSSD is a sector where activities that demand a lot of physical effort are carried out, and these activities are harmful to the health of the functionally-readapted professional. Almost half of these professionals disagree that the CSSD offers an adequate structure for the promotion of workers' health due to occupational and ergonomic risks. Almost all participants agree that the correct preparation of surgical trays can directly impact patient safety. Furthermore, there is a relationship between previous work in the CSSD and the ability to identify surgical tweezers by visual recognition, and between the time that the nursing professional previously worked at the hospital and the skills regarding the information contained in the conference folders for the preparation of surgical trays. The usual activities of the CSSD are considered exhaustive and with occupational hazards, which proves not to be suitable for the functionally-readapted professional. In this sense, the ideal would be for the CSSD to have professionals with skills free of serious comorbidities, and with knowledge of new technologies – which are fundamental interventions to be implemented by managers. Although CSSD professionals potentially acquire skills due to the length of service, it is noted that they need more favorable conditions for the verification of tweezers in the preparation of surgical trays. Managers' commitment to an internal health policy aimed at workers is necessary for the promotion of health and for the implementation of in-service training. In this sense, the role of society demanding the implementation of these measures is of great importance for valuing nursing professionals who work in the CSSD so that the paradigms of invisibility are broken, especially for those who are functionally-readapted people.

## Figures and Tables

**Table 1:** Participants' general characteristics (*n* = 72).

Variable	Number (%)

Age group	
20–30 years	4 (5.6%)
31–40 years	12 (16.7%)
41–50 years	23 (31.9%)
51–60 years	25 (34.7%)
>60 years	8 (11.1%)
Sex	
Male	18 (25%)
Female	54 (75%)
Marital status	
Single	26 (36.1%)
Married	35 (48.6%)
Stable union	9 (12.5%)
Divorced	1 (1.4%)
Widower	1 (1.4%)
Education	
Higher level	30 (41.7%)
High school	42 (58.3%)
Institutional bond	
Statutory	37 (51.4%)
Consolidation of labor laws	17 (23.6%)
Service provider	18 (25%)

Results are expressed as number (%).

**Table 2:** Professional profile of the participants (*n* = 72).

Variable	Number (%)

How long do you work in the hospital?	
1–10 years	22 (30.6%)
11–20 years	34 (47.2%)
21–30 years	0 (0%)
>31 years	36 (22.2%)
Have you ever worked on a CSSD before?	
Yes	29 (40.3%)
No	43 (59.7%)
Did you come to CSSD because of being re-adapted?	
Yes	22 (30.6%)
No	50 (69.4%)

CSSD: central sterile supply department.

**Table 3:** Distribution of participants' opinions regarding health risk, professional skills, and work environment in the central sterile supply department.

Variable	Number (%)
Some professionals consider CSSD activities to be easy to perform and require little effort	
Strongly disagree	0 (0%)
Somewhat disagree	2 2.8%)
Neutral	3 (4.2%)
Somewhat agree	24 (33.3%)
Strongly agree	43 (59.7%)
Some tasks performed in the CSSD can be considered harmful to the health of the functionally-readapted professional	
Strongly disagree	24 (33.3%)
Somewhat disagree	40 (55.6%)
Neutral	3 (4.2%)
Somewhat agree	5 (6.9%)
Strongly agree	0 (0%)
The CSSD offers an environment with an adequate structure to promote the health of the professional	
Strongly disagree	1 (1.4%)
Somewhat disagree	14 (19.4%)
Neutral	11 (15.3%)
Somewhat agree	30 (41.7%)
Strongly agree	16 (22.2%)
How do you rate performance in CSSD?	
Extremely difficult	1 (1.4%)
Difficult	12 (16.7%)
Moderate	41 (56.9%)
Easy	14 (19.4%)
Very easy	4 (5.6%)
How do you evaluate the information contained in the conference folders for preparing the surgical trays?	
Extremely difficult	2 (2.8%)
Difficult	7 (9.7%)
Moderate	41 (56.9%)
Easy	17 (23.6%)
Very easy	5 (7%)
Do your skills allow you to prepare a surgical tray without the use of conference folders?	
Very unlikely	7 (9.7%)
Unlikely	20 (27.8%)
Neutral	4 (5.6%)
Likely	23 (31.9%)
Very likely	18 (25%)
How do you classify the identification of surgical tweezers in the way they are numerically recorded?	
Extremely difficult	2 (2.8%)
Difficult	7 (9.7%)
Moderate	33 (45.8%)
Easy	26 (36.1%)
Very easy	4 (5.6%)
How do you classify the identification of surgical tweezers by visual recognition?	
Extremely difficult	0 (0%)
Difficult	9 (12.5%)
Moderate	31 (43.1%)
Easy	27 (37.5%)
Very easy	5 (6.9%)
Correct preparation of surgical trays contributes to patient safety	
Strongly disagree	0 (0%)
Somewhat disagree	0 (0%)
Neutral	1 (1.4%)
Somewhat agree	17 (23.6%)
Strongly agree	54 (75%)
In relation to other hospital sectors, the CSSD is a place with a high risk of contamination	
Strongly disagree	1 (1.4%)
Somewhat disagree	6 (8.3%)
Neutral	2 (2.8%)
Somewhat agree	36 (50%)
Strongly agree	27 (37.5%)
Actions aimed at preserving the environment must be implemented at the CSSD	
Strongly disagree	0 (0%)
Somewhat disagree	1 (1.4%)
Neutral	10 (13.9%)
Somewhat agree	38 (52.8%)
Strongly agree	23 (31.9%)
A sustainable product for identifying surgical tweezers improves their identification	
Very unlikely	2 (2.8%)
Unlikely	0 (0%)
Neutral	12 (16.7%)
Likely	34 (47.2%)
Very likely	24 (33.3%)

CSSD: central sterile supply department.

**Table 4:** Association between previous work at a central sterile supply department and professional ability.

Variable	Previous work in CSSD		*p*-value
	Yes	No	
	Number (%)	Number (%)	

How do you classify the identification of surgical tweezers by visual recognition?			
Difficult	4 (13.8%)	5 (11.6%)	**0.031**
Moderate	12 (41.4%)	19 (44.2%)	
Easy	8 (27.6%)	19 (44.2%)	
Very easy	5 (17.2%)	0 (0%)	

CSSD = central sterile supply department

**Table 5:** Association between working time at the hospital and professional ability.

Variable	Working time at the hospital			*p*-value
	1–10 years	11–20 years	>31 years	
	Number (%)	Number (%)	Number (%)	

How do you evaluate the information contained in the conference folders for preparing the surgical trays?				
Extremely difficult	0 (0%)	1 (2.9%)	1 (6.3%)	**0.001**
Difficult	0 (0%)	5 (14.7%)	2 (12.5%)	
Moderate	9 (40.9%)	22 (64.7%)	10 (62.4%)	
Easy	9 (40.9%)	6 (17.7%	2 (12.5%)	
Very easy	4 (18.2%)	0 (0%)	1 (6.3%)	

## Data Availability

The data used to support the findings of this study are available from the corresponding author upon reasonable request.

## Abbreviations

CSSD: Central sterile supply department
RSI: Repetitive strain injuries
WMSD: Work-related musculoskeletal disorders.
